# Autonomous Pointing Control of a Large Satellite Antenna Subject to Parametric Uncertainty

**DOI:** 10.3390/s17030560

**Published:** 2017-03-10

**Authors:** Shunan Wu, Yufei Liu, Gianmarco Radice, Shujun Tan

**Affiliations:** 1 School of Aeronautics and Astronautics, Dalian University of Technology, Dalian 116024, China; tansj@dlut.edu.cn; 2 Qian Xuesen Laboratory of Space Technology, China Academy of Space Technology, Beijing 100094, China; liuyufei@qxslab.cn; 3 School of Engineering, University of Glasgow, Glasgow G12 8QQ, UK; gianmarco.radice@glasgow.ac.uk

**Keywords:** attitude control, satellite, autonomous control, adaptive, antenna pointing

## Abstract

With the development of satellite mobile communications, large antennas are now widely used. The precise pointing of the antenna’s optical axis is essential for many space missions. This paper addresses the challenging problem of high-precision autonomous pointing control of a large satellite antenna. The pointing dynamics are firstly proposed. The proportional–derivative feedback and structural filter to perform pointing maneuvers and suppress antenna vibrations are then presented. An adaptive controller to estimate actual system frequencies in the presence of modal parameters uncertainty is proposed. In order to reduce periodic errors, the modified controllers, which include the proposed adaptive controller and an active disturbance rejection filter, are then developed. The system stability and robustness are analyzed and discussed in the frequency domain. Numerical results are finally provided, and the results have demonstrated that the proposed controllers have good autonomy and robustness.

## 1. Introduction

In recent years, the development of large orbiting structures to support Earth observation and mobile communication technology has been witnessed [1,2,3]. These applications generally require large, satellite-borne antennas. Due to the restrictions of the fairing of launch vehicles, the paradigm of large satellite antennas (LSAs) is being gradually implemented in both commercial and scientific missions such as Thuraya and National Reconnaissance Office Launch 26 (NROL-26) [4,5,6,7]. Since the antenna signal is weak during satellite on-orbit operations, the optical axis of the antenna should be continuously directed towards the target. High-precision pointing of the optical axis of an LSA is therefore extremely desirable.

Some research works have proposed different control methodologies to increase the LSA pointing precision. The LSA pointing control can be generally divided into two separate types: the first achieves the antenna pointing maneuver through the satellite attitude control and the second by means of an antenna pointing mechanism (APM) on board a satellite. The linear quadratic gaussian based control was proposed for large space antennas [8], and the effect of parameter variations was further discussed in [9]. A beacon-based pointing control design synthesis for a large flexible antenna was studied in [10], and a method of integrating the structure design into the design procedure was also addressed. Collocated and noncollocated pointing control strategies were proposed, and the results demonstrated that noncollocated pointing control is more accurate during both transient and steady-state modes [10]. The antenna pointing control strategy for tracking and data relay satellites was also studied [11], in which the antenna pointing control concepts were described, and an on-board autonomous control scheme, including acquisition and autotrack modes, was proposed. An active disturbance rejection control for the antenna pointing control of a large flexible satellite system was proposed in [12], and the inner and outer loops of the control system were studied to improve pointing accuracy and rotation speed. The attitude control system on the Engineering Test Satellite-VIII, which has two large deployable antennas, was studied in [13]. The phase stabilization control for low mode frequencies and small damping, and the gain stabilization control for higher mode frequencies were respectively designed. Classical control design techniques were proposed for the Tracking and Data Relay Satellite antenna. The loop shaping provides good single loop stability margins, and multiloop stability margin analysis verifies stability robustness against sensor parametric uncertainties, modal frequency shifts and gain/phase variations [14]. An H∞ control approach, to achieve pointing control design of a flexible spacecraft antenna, was addressed in [15]. A proportional–derivative (PD) plus structural filter was designed to improve pointing accuracy and suppress vibration, and the frequency-domain method was used to analyze system performance [16].

All the above-mentioned works utilize satellite attitude maneuvers to perform large antenna pointing control. Meanwhile, an APM-based approach, to perform pointing control of LSA, was proposed in [17,18]. The APM, which can drive antenna maneuvers and then correct the pointing errors, is installed on the satellite body. If the antenna is relatively small and its structural frequency is high, the APM-based approach can be used for a high-performance antenna pointing control. However, if the antenna is large with high moments of inertia and low structural frequency, the fundamental frequency of the whole satellite–antenna system will be mainly driven by the antenna. In this case, the bandwidth of APM-based pointing control loop is then not much higher than that of the satellite attitude control loop, which will thus lead to complicated attitude dynamic coupling problems. In this scenario, satellite attitude stabilization could be significantly disturbed during APM maneuvers. Correcting the pointing error of the LSA optical axis by means of the satellite attitude control is therefore more practical [19,20,21,22]. 

The main factors that influence on-orbit pointing precision of LSA are twofold [16]. On the one hand, the LSA has very large dimensions and low stiffness, and the structural vibrations that may arise due to disturbances could seriously affect pointing and even system stability. On the other hand, the flexible satellite and LSA are subject to weight loss, thermal radiation and temperature variation in space. The modal parameters, which are uncertain or even time-varying, cannot not be known exactly. Besides, the thermal deformation error due to solar radiation, installation error and the deployment error also affect LSA pointing. All the above issues make it difficult to achieve high precision for LSA optical axis pointing. Besides, current space missions always require autonomy and intelligence, such as on-orbit identification and intelligent control. That means intelligent control techniques are applied in spacecraft systems, which could make the spacecraft perform on-orbit operations autonomously without the support of the ground station and astronauts. Consequently, it is imperative to take the stability of closed-loop system, parametric uncertainty and pointing errors into consideration simultaneously, and then develop a new intelligent control algorithm to achieve autonomous pointing maneuvers. The adaptive control approach provides an ideal solution to deal with this problem since it can handle online estimation for uncertain and unknown system parameters.

To address these challenges, frequency-domain methodology is used to design the autonomous pointing controllers in this paper. The proportional–derivative feedback and structural filter, to perform pointing maneuvers and suppress antenna vibrations, are firstly proposed. A modified adaptive controller, based on above controller and adaptive filter technique is then developed in the presence of modal parameter uncertainty. The active disturbance rejection filter is finally designed and integrated into the closed-loop system. The novelty lies in that the proposed autonomous controller could estimate in orbit, compensate the uncertain modal parameters and decrease the periodic pointing error simultaneously. The proposed control approach can avoid excessive complexity of the control laws, and reduce the dependency of the controller on the knowledge of the system parameters.

## 2. LSA Pointing Dynamics

**Figure 1:**
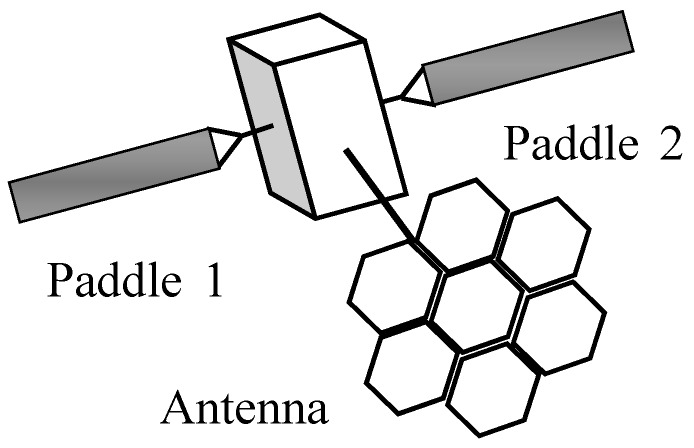
A satellite with flexible appendages.

The LSA is fixed on the satellite body, as shown in Figure 1, and the attitude dynamics include two components: the attitude dynamic model of the flexible satellite and the antenna pointing in the satellite body coordinate system. The attitude dynamics of flexible satellite is governed by the following differential equations [23,24,25]:

(1) $$\{\begin{array}{c} I\;\ddot{\theta }+\sum_{i=1}^{m}B_{roti}{\ddot{\eta }}_{i}=T+T_{d}\\ {\ddot{\eta }}_{i}+2\varsigma_{i}\Lambda_{i}{\dot{\eta }}_{i}+{\Lambda_{i}}^{2}\eta_{i}+\sum_{i=1}^{m}{B_{roti}}^{T}\ddot{\theta }=0\\ i=1,2,...,m \end{array}$$

where I is the inertia matrix, θ is the vector of the satellite attitude angles, $B_{roti}$ is the rotational coupling coefficient of the *i*th flexible appendage, $\eta_{i}$ is the modal coordinate, $\varsigma_{i}$ is the modal damping ratio, $\Lambda_{i}$ is the modal frequency, and T and $T_{d}$ are the control torque and disturbance torque respectively. According to Craig–Bampton mode synthesis, the internal degree of freedom (DOF) of LSA is given by:

(2) $$X_{Ia}=\phi_{IJa}X_{Ja}+\phi_{Ia}\eta_{a}$$

where $\phi_{IJa}$ is the constrained modal matrix, $\phi_{Ia}$ is the main modal matrix, $X_{Ja}$ represents interface DOF and $\eta_{a}$ is the LSA modal coordinate. Let i,j,k represent the LSA pointing DOF and $L=\left[L_{i},\;L_{j},\;L_{k}\right]$ denotes the pointing DOF matrix. Then, the pointing DOF in the satellite body coordinate system is [9,10]:

(3) $$\theta_{a}=LX_{Ia}=L\phi_{IJa}X_{Ja}+L\phi_{Ia}\eta_{a}=\theta +L\phi_{Ia}\eta_{a}$$

For each LSA pointing DOF, there is $L_{\nu }=\left[l_{\nu 1},\;l_{\nu 2},\;l_{\nu 3},\ldots l_{\nu n}\right]\;\nu =i,j,k$, where $l_{\nu 1}\ldots l_{\nu n}$ represents the weight of each DOF in antenna pointing. Considering the thermal deformation error $\theta_{the}$, the installation error $\theta_{ins}$ and the deployment error $\theta_{rep}$, Equation (3) can be rewritten as:

(4) $$\theta_{a}=\theta +L\phi_{Ia}\eta_{a}+\theta_{the}+\theta_{ins}+\theta_{rep}$$

where $\theta_{a}={\left[\theta_{ax}\;\theta_{ay}\;\theta_{az}\right]}^{T}$. Combining Equations (1) and (4) yields the following attitude dynamics with antenna pointing:

(5) $$\{\begin{array}{c} I\;\ddot{\theta }+\sum_{i=1}^{m}B_{roti}{\ddot{\eta }}_{i}=T+T_{d}\\ {\ddot{\eta }}_{i}+2\varsigma_{i}\Lambda_{i}{\dot{\eta }}_{i}+{\Lambda_{i}}^{2}\eta_{i}+\sum_{i=1}^{m}{B_{roti}}^{T}\ddot{\theta }=0\\ \begin{array}{c} i=1,2,...,m\\ \theta_{a}=\theta +L\phi_{Ia}\eta_{a}+\theta_{the}+\theta_{ins}+\theta_{rep} \end{array} \end{array}$$

Then, the LSA pointing dynamics are given by:

(6) $$\{\begin{array}{c} I\;{\ddot{\theta }}_{a}+\sum_{i=1}^{m}B_{\mathrm{roti}}{\ddot{\eta }}_{i}-I\;{\ddot{\theta }}_{\Delta }=T+T_{d}\\ {\ddot{\eta }}_{i}+2\varsigma_{i}\Lambda_{i}{\dot{\eta }}_{i}+{\Lambda_{i}}^{2}\eta_{i}+\sum_{i=1}^{m}{B_{\mathrm{roti}}}^{T}\ddot{\theta }=0\\ i=1,2,...,m \end{array}$$

where $\theta_{\Delta }=L\phi_{\mathrm{Ia}}\eta_{a}+\theta_{\mathrm{the}}+\theta_{\mathrm{ins}}+\theta_{\mathrm{rep}}$.

**Remark** **1.** *If we define the pointing angle* $\phi ={\left[\varphi \;\vartheta \;\sigma \right]}^{T}$ *in the antenna body coordinate system, then* ϕ *can be obtained by:*

(7) $$\phi =C\theta_{a}$$

*where* C *is the transformation matrix from the satellite body coordinate system to the antenna body coordinate system. The rotation order is Z-X-Y and rotation angles are* $\theta_{z}$*,* $\theta_{x}$ *and* $\theta_{y}$*; then,* C *is given by:*

(8) $$C=\left[\begin{array}{ccc} c\theta_{y}c\theta_{z}-s\theta_{y}s\theta_{x}s\theta_{z} & c\theta_{y}s\theta_{z}+s\theta_{y}s\theta_{x}c\theta_{z} & -s\theta_{y}c\theta_{x}\\ -s\theta_{z}c\theta_{x} & c\theta_{z}c\theta_{x} & s\theta_{x}\\ s\theta_{y}c\theta_{z}+c\theta_{y}s\theta_{x}s\theta_{z} & s\theta_{y}s\theta_{z}-c\theta_{y}s\theta_{x}c\theta_{z} & c\theta_{y}c\theta_{x} \end{array}\right]$$

*where c represents cosine function and s denotes sine function.*

## 3. Autonomous Controller Design

The pointing angles of the LSA are measured by an appropriate sensor, and this measured value can be used as feedback to correct pointing errors, considered as the noncollocated control. As can be seen in Equation (6), there exists coupling among satellite attitude angles, antenna pointing angles and control torques. A commonly-used method in practice is to diagonalize I and $B_{roti}$ through satellite structure design, then the satellite with large antenna is designed as three-axis decoupling. The autonomous pointing control of LSA pitch-axis, which is the most representative, is therefore proposed in this paper; others are similar with respect to pitch axis and are thus omitted here. 

The design process of the autonomous pointing controller is addressed below. PD feedback plus a structural filter to perform pointing maneuvers and suppress antenna vibration are firstly presented in this section. Based on the controller, a modified adaptive controller is proposed subject to parametric uncertainty and pointing errors. Furthermore, the frequency-domain analysis technique is employed to design the control system, which is more practical in engineering problems. The proposed control approach has a simple structure, low orders and clear physical significance, which therefore avoids excessive complexity and provides a possible solution for engineering projects.

### 3.1. PD Plus Structural Filter Design

It is assumed that the satellite has only one flexible appendage, the LSA. We firstly propose a pointing controller, and the transfer function of the satellite pitch axis can be then given by:

(9) $$\frac{\theta_{y}(s)}{T_{y}(s)}=\frac{1}{I_{y}s^{2}\left(1-\sum_{j}\frac{k_{j}s^{2}}{s^{2}+2\varsigma_{j}\Lambda_{j}s+{\Lambda_{j}}^{2}}\right)}$$

where $\theta_{y}$ and $T_{y}$ are the satellite pitch angle and pitch-axis control torque respectively, $I_{y}$ is the pitch inertia, s is the Laplace transform variable, $k_{j}$ is the *j*th modal gain of LSA, $\varsigma_{j}$ and $\Lambda_{j}$ are the modal damping ratio and the modal frequency of LSA pitch axis. The LSA pointing can be transformed as:

(10) $$\frac{\theta_{ay}}{\theta_{y}}=1-s^{2}\sum_{j}\frac{L_{y}\phi_{Iay}B_{rotj}}{s^{2}+2\varsigma_{j}\Lambda_{j}s+{\Lambda_{j}}^{2}}$$

where $\theta_{ay}$ is the LSA pitch pointing angle. According to Equations (9) and (10), the transfer function of LSA pitch pointing in the satellite body coordinate system is given by:

(11) $$\frac{\theta_{ay}}{T_{y}}=\frac{1-s^{2}\sum_{j}\frac{L_{y}\phi_{Iay}B_{rotj}}{s^{2}+2\varsigma_{j}\Lambda_{j}s+{\Lambda_{j}}^{2}}}{I_{y}s^{2}\left(1-\sum_{j}\frac{k_{j}s^{2}}{s^{2}+2\varsigma_{j}\Lambda_{j}s+{\Lambda_{j}}^{2}}\right)}$$

where the subscript y represents pitch axis variables of satellite and LSA. 

**Figure 2:**
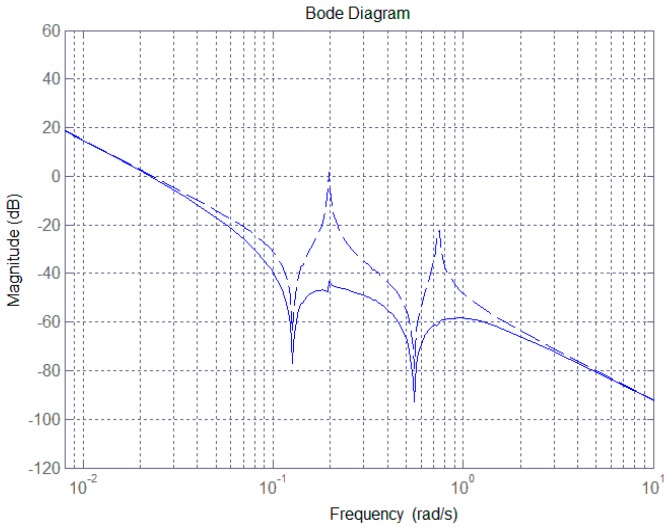
Bode magnitude diagram of satellite and antenna pitch axis.

For the system presented in Equation (11), the conventional PD feedback can stabilize the antenna pointing control system, but cannot effectively suppress structural vibrations of the antenna. Figure 2 shows the open-loop Bode diagram of the LSA pointing control system (dashed line). As can be seen, the magnitude is amplified and exhibits a large discontinuity at the bending frequencies of 0.198 rad/s and 0.745 rad/s corresponding to unstable poles for the control system. This arises from the vibrations of the antenna structure, which can seriously affect pointing precision and wreck system stability. 

The structural filter (SF) can provide a possible solution to suppress the vibration, and improve system performance. The SF can be realized from a second-order filter, represented as:

(12) $$\frac{s^{2}/{\omega_{z}}^{2}+2\varsigma_{z}s/\omega_{z}+1}{s^{2}/{\omega_{p}}^{2}+2\varsigma_{p}s/\omega_{p}+1}$$

where $\omega_{z}$ and $\omega_{p}$ are the frequencies of SF zeros and poles, and $\varsigma_{z}$ and $\varsigma_{p}$ are the damping ratios. For different choices of $\omega_{z}$, $\omega_{p}$, $\varsigma_{z}$ and $\varsigma_{p}$, different filters can be achieved. The principle and design process of the SF have been clearly investigated by the authors of [16]. The notch filter is a kind of gain-stable filter, and can be used to suppress structure vibration for LSA pointing control. Let the frequencies of SF zeros equal to the frequencies of unstable poles, then the vibration caused by the unstable poles could be suppressed in the closed-loop system. 

The inertia matrix of the pitch-axis is $I_{y}=18,050\;\mathrm{kg}\cdot m^{2}$, and other representative parameters are given in Table 1 [16]. As shown in Figure 2, the magnitude jumps at the frequencies of 0.198 rad/s and 0.745 rad/s. Then controller is therefore given by:

(13) $$T_{y}(s)=-(520s+10)\cdot \frac{25.5s^{2}+0.1s+1}{25.5s^{2}+18.2s+1}\cdot \frac{1.83s^{2}+0.01s+1}{1.83s^{2}+2s+1}\cdot \psi (s)$$

where ψ(s) denotes the pitch pointing angle error, the subscript j represents the modal order, and the first four-order modal parameters are chosen. The magnitude plot of open loop system with controller from Equation (13) is shown in Figure 2 (solid line). Obviously, the magnitude at the frequencies of 0.198 rad/s and 0.745 rad/s is well reduced through introducing a notch filter into feedback loop. The SF can effectively remove the vibration signals embedded in the attitude dynamics.

**Remark** **2.** *The closed-loop system with controller from Equation (13) has a gain margin of 60.2 dB and phase margin of 155 deg, which is therefore stable. For the proposed notch filter presented in the controller from Equation (13), the maximum magnitude gain can be obtained by* $-20\log_{10}\frac{\varsigma_{z}}{\varsigma_{p}}$ *which occurs at* $\omega_{z}$*. The filter damping ratios will also determine the effective notch region and system settling time*.

### 3.2. Adaptive Filter Control Design

**Figure 3:**
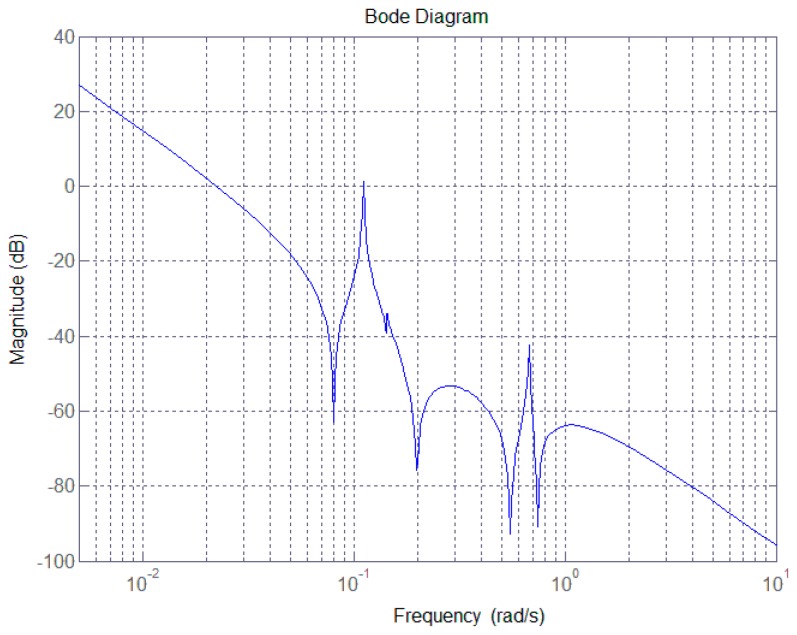
Bode magnitude diagram subject to modal parameter uncertainty.

In practice, the flexible satellite and LSA will be subject to weight loss, thermal radiation and temperature variations once in orbit. The modal parameters may well be uncertain, and even time-varying, which would render achieving the actual bending frequency extremely difficult. If the first-order modal parameters presented in Table 1 were to change, for example in the case that the values of $\Lambda_{1}$, $\varsigma_{1}$ and $B_{rot1}$ changed to 0.082, 0.004 and 93, respectively, and the controller from Equation (13) was still adopted in the control loop to perform pointing maneuvers, then the corresponding Bode magnitude plot is that shown in Figure 3. 

**Figure 4:**
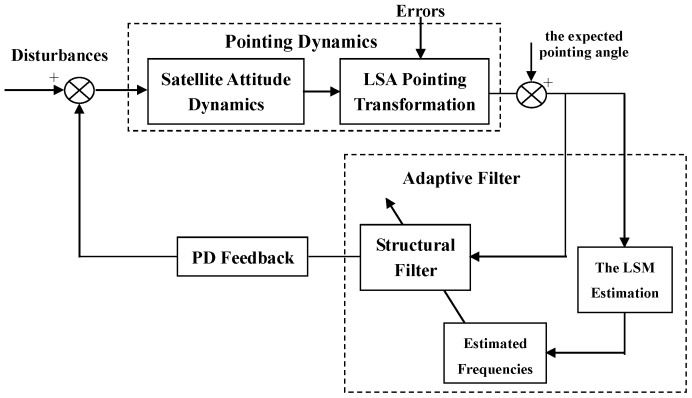
Adaptive filter control system. PD: proportional–derivative; LSM: least squares method; LSA: large satellite antenna.

As can be seen, the magnitude presents significant discontinuity at the bending frequencies of 0.11 rad/s and 0.68 rad/s although the controller in Equation (13) is included in the system. This is because a particular SF can only remove particular bending vibration signals. Thus, in order to meet the principle of the same SF frequency as that of the bending vibration, an adaptive algorithm that estimates the actual system bending frequency is required. Based on the estimated values, the controller from Equation (13) is then redesigned. The block diagram of the LSA pointing control system is shown in Figure 4.

The least squares method (LSM) is proposed to estimate the actual bending frequency. The transfer function from Equation (11) is discretized, and its difference equation can be written as:

(14) $$A(z^{-1})y(k)=B(z^{-1})u(k-d)+\varepsilon (k)$$

where $A(z^{-1}),\;B(z^{-1})$ are discrete unit operator polynomials, ε(k) represents the external disturbances and d denotes the order of delay links. $A(z^{-1})$ and $B(z^{-1})$ can be developed as series expansions:

(15) $$A(z^{-1})=1+a_{1}z^{-1}+a_{2}z^{-2}...+a_{n_{a}}z^{-n_{a}}$$

(16) $$B(z^{-1})=b_{0}+b_{1}z^{-1}+b_{2}z^{-2}...+b_{n_{b}}z^{-n_{b}}$$

where $n_{a}$ and $n_{b}$ denote the system orders, while $a_{i}$ and $b_{i}$ represent the parameters to be estimated. Equation (14) is rewritten as:

(17) $$\begin{array}{lll} y(k) & = & -a_{1}y(k-1)-a_{n_{a}}y(k-n_{a})\\ & & +b_{0}u(k-d)+b_{n_{b}}u(k-d-n_{b})+\varepsilon (k)\\ & = & \varphi^{T}(k)\delta +\varepsilon (k) \end{array}$$

where φ(k) and δ are the observation vector of system inputs and outputs and the coefficient matrix, and are respectively given by:

(18) $$\varphi (k)={\left[-y(k-1),...,-y(k-n_{n_{a}}),u(k-d),...,u(k-d-n_{n_{a}})\right]}^{T}$$

(19) $$\delta ={\left[a_{1},...,a_{n_{a}},b_{0},...,b_{n_{b}}\right]}^{T}$$

The quadratic performance index is defined as:

(20) $$\begin{array}{c} J=\varepsilon^{T}\varepsilon ={\left(\varphi^{T}\hat{\delta }-y\right)}^{T}\left(\varphi^{T}\hat{\delta }-y\right)\\ ={\hat{\delta }}^{T}\left(\varphi^{T}\varphi \right)\hat{\delta }-2y^{T}\varphi^{T}\hat{\delta }+y^{T}y \end{array}$$

where $\hat{\delta }$ is the estimated value of δ. We can minimize J to obtain $\hat{\delta }$, namely $\frac{\partial J}{\partial \hat{\delta }}=0$. $\hat{\delta }$ can be then obtained by the batch processing, and there exists:

(21) $$\hat{\delta }={\left(\Phi^{T}\Phi \right)}^{-1}\Phi^{T}Y$$

where $\Phi ={\left[\varphi^{T}(1)\;\varphi^{T}(2)\;,...,\;\varphi^{T}(n)\right]}^{T}$ and $Y={\left[y(1),\;y(2)\;,...,\;y(n)\right]}^{T}$. Hence, we can solve Equations (15) and (16) for $a_{i}$, $b_{i}$. The actual bending frequency can be calculated by:

(22) $$\omega^{*}=\frac{1}{T_{s}}\sqrt{\ln z_{r}\ln z_{r}^{*}}$$

where $T_{s}$ is the sampling time, $z_{r}$ and $z_{r}^{*}$ are conjugate poles with maximum imaginary part of discrete system as shown in Equation (14). Considering the first order modal parameter uncertainty mentioned above, we can obtain $z_{r1}=-0.5041+0.816i$, ${z_{r1}}^{*}=-0.5041-0.816i$, $z_{r2}=-0.7860+0.6081i$, ${z_{r2}}^{*}=-0.786-0.6081i$ and ${\omega_{1}}^{*}=0.11$ rad/s, ${\omega_{2}}^{*}=0.66$ rad/s. Then the controller as shown in Equation (13) can be redesigned. The technical process can be readily accomplished by following the line in the above section. Then, the adaptive-filter-based pointing controller is finally given by:

(23) $$T_{y}(s)=-(520s+10)\cdot \frac{82.645s^{2}+0.1s+1}{82.645s^{2}+18.2s+1}\cdot \frac{2.3s^{2}+0.01s+1}{2.3s^{2}+2s+1}\cdot \psi (s)$$

Only the uncertainties of the first order modal parameters are discussed above. If the uncertainties on the modal parameters of other modes are also considered, such as the second-order modal parameters, the actual bending frequencies could change and the magnitude will present discontinuity as well. Then, the adaptive filter control is designed as similar to the above process, and is thus omitted here. 

### 3.3. Active Disturbance Rejection Filter

**Figure 5:**
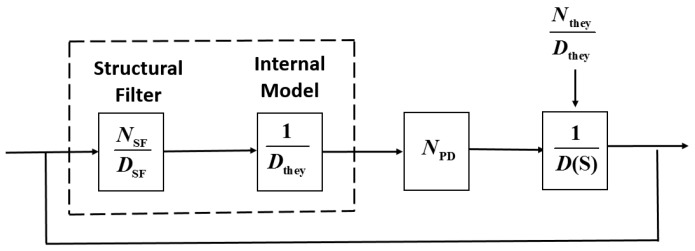
Active disturbance rejection control system. SF: structural filter.

Thermal deformation errors, installation errors and deployment errors can all negatively affect the accuracy and stability of LSA pointing. The installation error and the deployment error can generally be assumed to be constant, and thus can be compensated through precise calibration and adding an integrator in a closed-loop system. During in-orbit operations, solar radiation pressure may periodically lead to antenna thermal deformation, which brings about periodic errors in the LSA pointing. After successful stabilization of the satellite and flexible LSA, to decrease the periodic pointing error, the active disturbance rejection technique is introduced into the feedback loop. The solar radiation-induced periodic error, $\theta_{they}$, is firstly modeled as:

(24) $$\theta_{they}=\theta_{1}\sin(p_{1}t+\phi_{\;1})$$

where $\theta_{1}$ and $\phi_{1}$ are the unknown amplitude and phase angle, while $p_{1}$ denotes the known frequency. In general, the error $\theta_{they}$ can be described by a Laplace transformation:

(25) $$\theta_{they}(s)=\frac{N_{they}(s)}{D_{they}(s)}$$

where $N_{they}(s)$ is arbitrary, while the roots of $D_{they}(s)$ correspond to the frequencies at which periodic excitation takes place. The active disturbance rejection filter in the control loop provides a solution for effective cancellation of the poles of $\theta_{they}(s)$, which is on the basis of the internal model principle. As shown in Figure 5, the closed-loop transfer function is:

(26) $$y(s)=\frac{D_{SF}(s)D_{\mathrm{they}}(s)}{D_{they}(s)D_{SF}(s)D(s)+N_{SF}(s)N_{PD}(s)}\frac{N_{\mathrm{they}}(s)}{D_{\mathrm{they}}(s)}$$

The presence of $1/D_{\mathrm{they}}(s)$ in the control loop results in the effective cancellation of the poles of $\theta_{they}(s)$, provided that no root of $D_{\mathrm{they}}(s)$ is a zero of the system transfer function. Then, an active disturbance rejection filter can be designed that has proper transfer function and uses $1/D_{\mathrm{they}}(s)$. Besides, a proper numerator is chosen to go with $1/D_{\mathrm{they}}(s)$, which is of the same order as $D_{\mathrm{they}}(s)$, and as such that there is a zero for each pole.

The active disturbance rejection filter can employ a zero-pole combination, and is given by:

(27) $$\frac{s^{2}/{z_{1}}^{2}+2\xi_{z}s/z_{1}+1}{s^{2}/{p_{1}}^{2}+1}$$

where $z_{1}$ and $p_{1}$ are a pair of zero poles, and $\xi_{z}$ is a gain. The modified controller is thus given by:

(28) $$T_{z}(s)=-\left(K_{d}s+K_{p}\right)\cdot \frac{s^{2}/{\omega_{z}}^{2}+2\varsigma_{z}s/\omega_{z}+1}{s^{2}/{\omega_{p}}^{2}+2\varsigma_{p}s/\omega_{p}+1}\cdot \frac{s^{2}/{z_{1}}^{2}+2\xi_{z}s/z_{1}+1}{s^{2}/{p_{1}}^{2}+1}\cdot \psi (s)$$

Equation (28) can then be rewritten as:

(29) $$T_{z}(s)=-\left(K_{d}s+K_{p}\right)\cdot \frac{s^{2}/{\omega_{z}}^{2}+D_{1z}s+1}{s^{2}/{\omega_{p}}^{2}+D_{2z}s+1}\cdot \frac{T_{z1}s^{2}+D_{z1}s+1}{s^{2}/{p_{1}}^{2}+1}\cdot \psi (s)\;$$

where $K_{d}$, $K_{p}$, $D_{1z}$, $D_{2z}$, $T_{z1}$ and $D_{z1}$ are the design parameters. For the solar radiation induced error $p_{1}=0.01\;\mathrm{rad}/s$ [16], hence, the modified autonomous pointing controller is governed by the following equations:

(30) $$\begin{array}{c} T_{z}(s)=-(520s+10)\cdot \frac{1\times 10^{6}s^{2}+200s+1}{1\times 10^{6}s^{2}+1}\cdot F(s)\psi (s)\\ F(s)=\frac{25.5s^{2}+0.1s+1}{25.5s^{2}+18.2s+1}\cdot \frac{1.83s^{2}+0.01s+1}{1.83s^{2}+2s+1} \end{array}$$

**Remark** **3.** *The separation between the zero and the pole affects the setting time of the closed-loop system. Generally, the larger the separation is, the shorter the settling time will be. This is a consequence of the position of the closed-loop eigenvalue corresponding to the zero-pole. As the separation is increased, the eigenvalue is pushed farther to the left, speeding up the response time of the rejection filter. The separation however also influences the gain-phase characteristics of the system. The magnitude of the gain actually increases with the separation between zero and pole. A proper* $z_{1}$ *and* $p_{1}$ *should be chosen to balance the settling time and the stability of the closed-loop system.*

**Remark** **4.** *If the constant errors are further considered in the closed-loop system, the controller from Equation (30) can be rewritten as:*

(31) $$T_{z}(s)=-(520s+10+\frac{0.02}{s})\cdot \frac{1\times 10^{6}s^{2}+200s+1}{1\times 10^{6}s^{2}+1}\cdot F(s)\psi (s)$$

*where an integrator* $\frac{0.02}{s}$ *is added to the closed-loop system, and then a proportional-integral-derivative controller is achieved. The integrator and rejection filter have different effects, where the integrator is used to decrease the steady-state constant error and rejection filter can decrease periodic error of the closed-loop system. However, an integrator could also destroy dynamic performance of closed-loop system.*

## 4. Numerical Results

**Figure 6:**
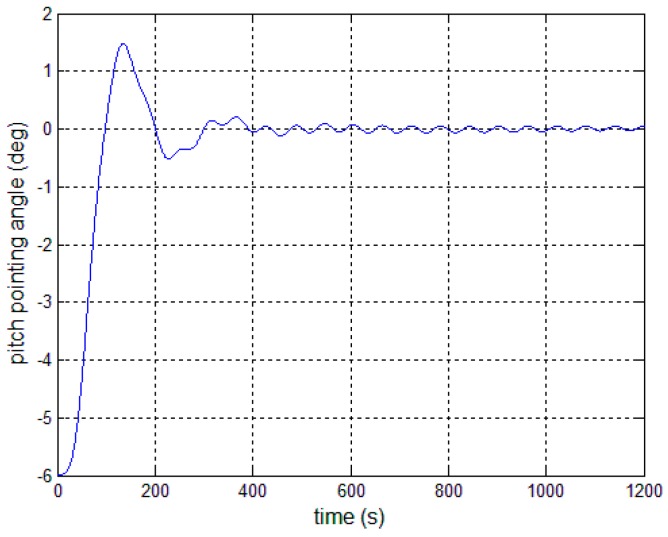
Antenna pitch angle error—controller (13).

**Figure 7:**
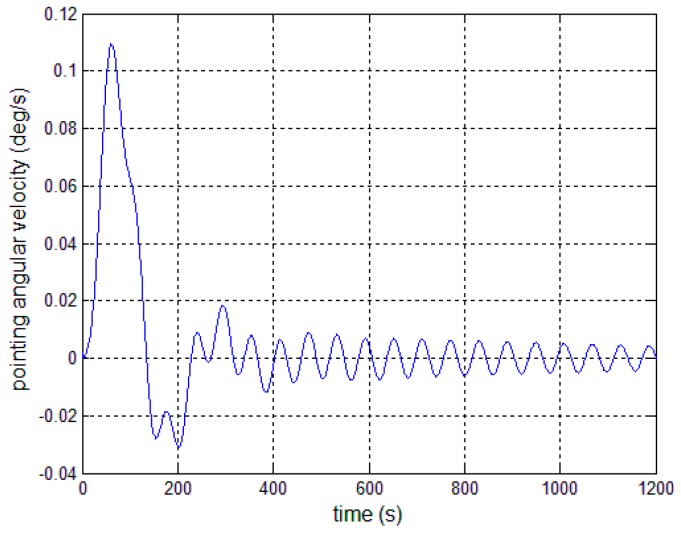
Antenna pitch angular velocity error—controller (13).

In this section, numerical results showing the performance of the proposed autonomous control algorithms are presented. The satellite parameters are given in Table 1, and the first four-order modal parameters are chosen. The periodic error is firstly given as 0.3° sin(0.01*t*). The controllers are given by Equations (13), (23) and (30). The expected antenna pitch angle is 6°, the initial pointing angle and angular velocity are both 0, and the installation error and the deployment error of pitch angle are both set at 0.001°. Figure 6 and Figure 7 present the outcome of the performance of the controller in Equation (13) subject to the first-order modal parameter uncertainty. As can be seen, the pointing angle error and the angular velocity error can converge to ±0.1° and ±0.01°/s, while there obviously exists periodic oscillation along the pitch axis. This demonstrates that a particular SF is only effective for the particular bending vibration signal. Once the modal parameters change, the performance of the closed-loop control system becomes worse.

**Figure 8:**
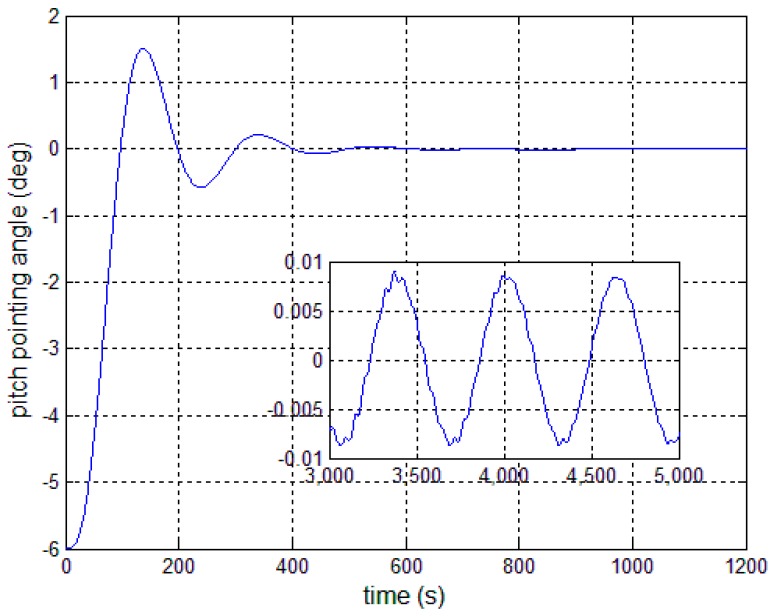
Antenna pitch angle error—controller, Equation (23).

**Figure 9:**
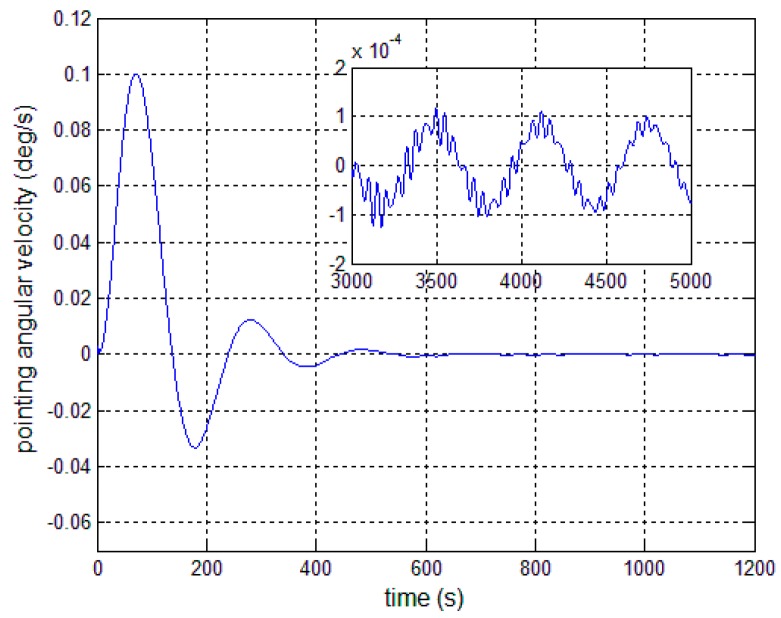
Antenna pitch angular velocity error—controller, Equation (23).

**Figure 10:**
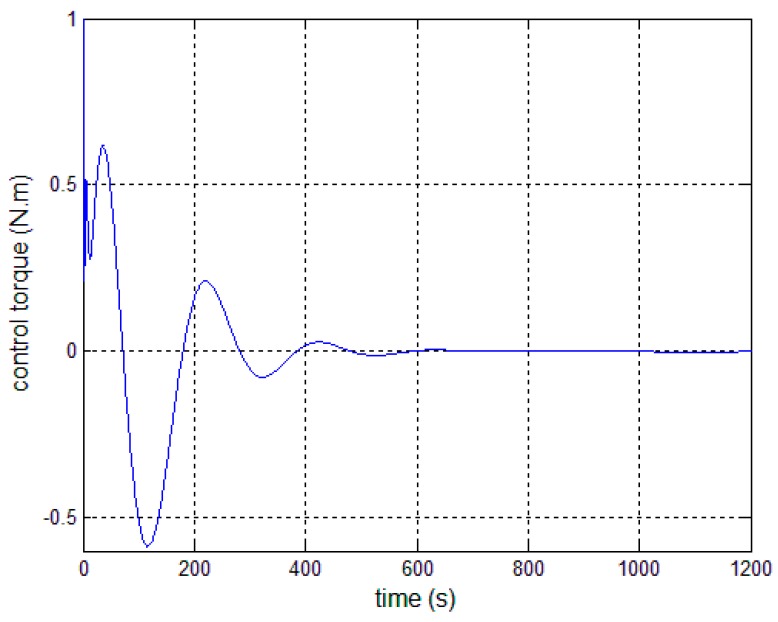
Control torque—controller, Equation (23).

**Figure 11:**
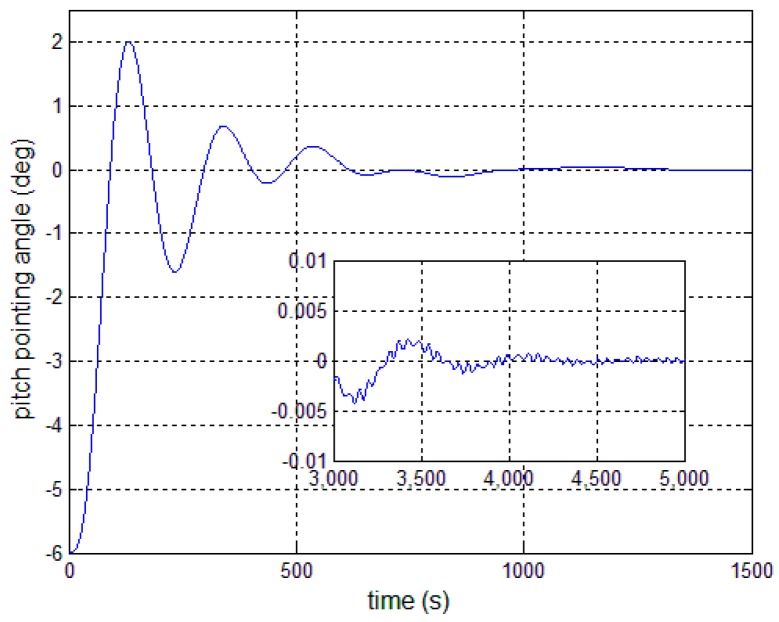
Antenna pitch angle error—controller, Equation (30).

**Figure 12:**
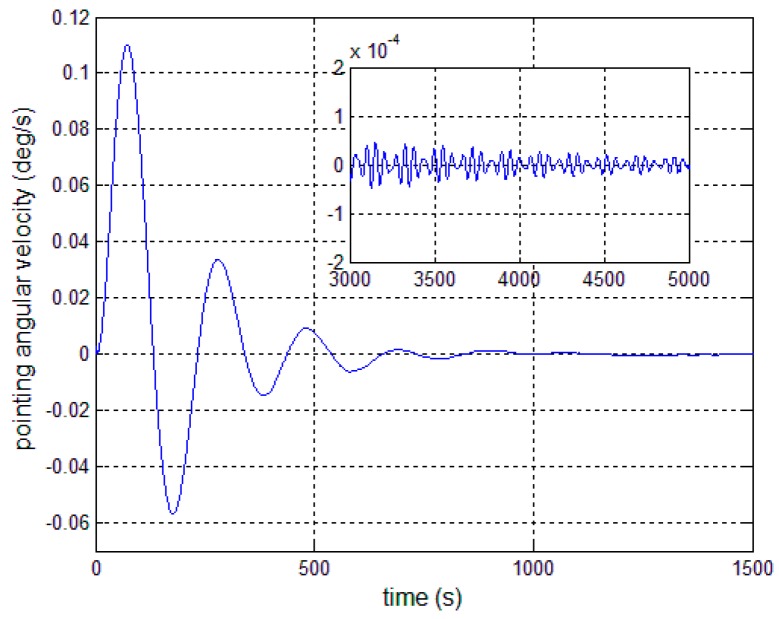
Antenna pitch angular velocity error—controller, Equation (30).

**Figure 13:**
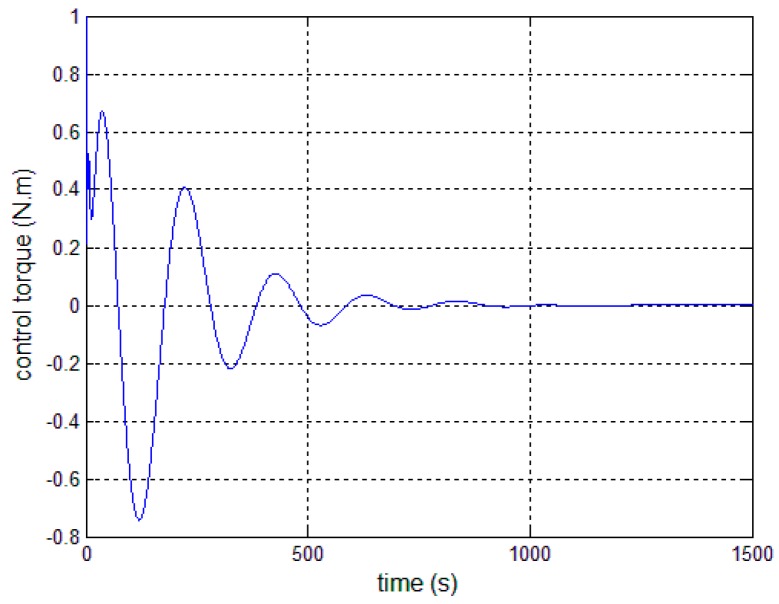
Control torque—controller, Equation (30).

**Figure 14:**
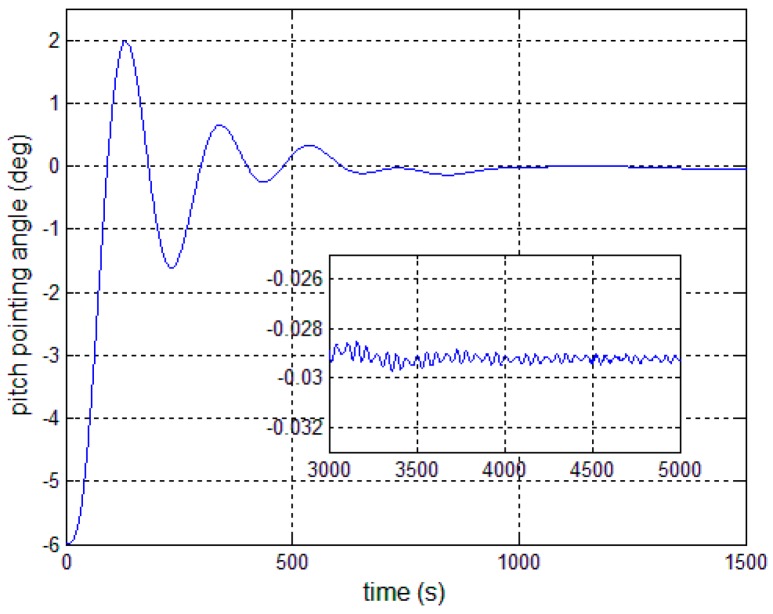
Antenna pitch angle error—controller, Equation (30).

**Figure 15:**
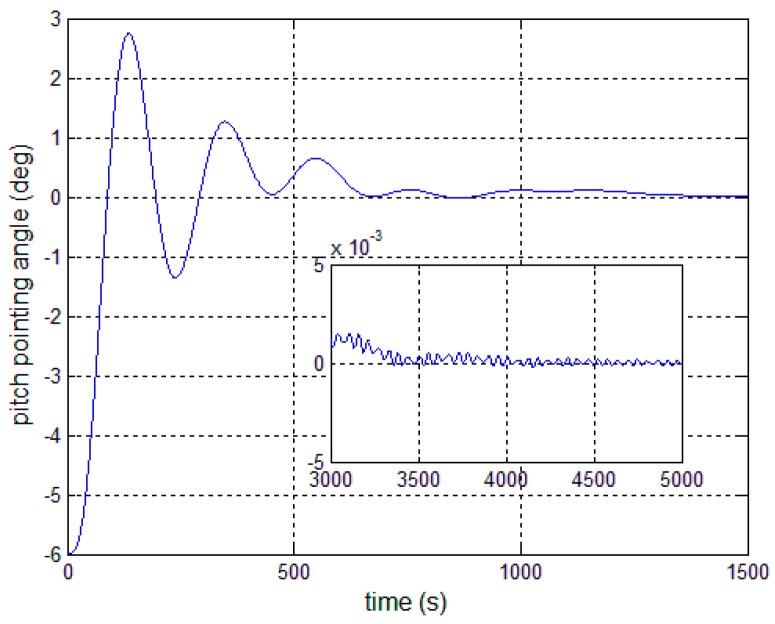
Antenna pitch angle error—controller, Equation (31).

Figure 8, Figure 9 and Figure 10 show the pitch angle error, angular velocity error and control torque resulting from the implementation of controller in Equation (23). The steady-state errors converge to ±0.01° and ±1 × 10^−4^°/s in approximately 600 s. However, the steady-state errors appear to have periodic oscillations. Comparing Figure 8 and Figure 9 with Figure 6 and Figure 7, it can be seen that the proposed adaptive filter controller (23) provides a better pointing accuracy and stability in the presence of modal parameter uncertainty. This is because an LSM is employed to estimate the actual frequencies and the SF is therefore redesigned. The numerical results of implementing the controller shown in Equation (30) are shown in Figure 11, Figure 12 and Figure 13. As can be seen, the pitch pointing errors are decreased, namely less than to ±0.01° and ±1 × 10^−4^°/s in approximately 1000 s, with a decrease by one order of magnitude further in time as shown in sub-plots. This means that the periodic error is effectively compensated by the rejection filter. Figure 10 and Figure 13 present the control torques of controllers in Equations (23) and (30), which have similar amplitude. The pointing errors include two components: the constant error and the periodic error, such as 0.3° sin(0.01*t*) + 0.29°. The pointing errors of pitch axis by implementing controllers in Equations (30) and (31) are then shown in Figure 14 and Figure 15 respectively. As can be seen, the integrator in Equation (31) can decrease the steady-state constant error. It can be therefore concluded that different control schemes are able to reduce different pointing errors, and then finally increase the pointing accuracy and robustness. 

## 5. Conclusions

The autonomous pointing problem of a large satellite antenna, which is achieved through satellite attitude maneuvers, is addressed in this paper. The LSA pointing dynamics are firstly proposed. The LSM-based adaptive controllers and active disturbance rejection filter are then respectively implemented in the presence of modal parameter uncertainty and pointing errors. It should be noted that the modal parameter uncertainty could lead to poorer performance for LSA pointing control. This is because a particular SF can only remove particular bending vibration signals. The LSM can effectively estimate the actual bending frequency, and then the LSM-based adaptive controller can increase pointing accuracy and stability. The active disturbance rejection filter is designed in the modified controller, which can further reduce periodic pointing errors. To deal with constant error, the integrator provides a realizable solution for a closed-loop system. Numerical results are finally presented to show that the proposed autonomous controllers are effective and simple, which makes them easier to implement in real-time applications. For the future work, the modified least squares methods, to further improve the estimation performance and efficiency of actual frequency, could be investigated for autonomous controller design. 

## Figures and Tables

**Table 1:** The modal parameters of pitch axis.

*j*	$\Lambda_{j}$	$B_{rotj}$	$\varsigma_{j}$	$L_{y}\phi_{\mathrm{Iay}}$
1	0.1270	103.491	0.005	$1.01\times 10^{-2}$
2	0.1420	6.485	0.005	$5.51\times 10^{-4}$
3	0.3401	2.667	0.005	$4.433\times 10^{-4}$
4	0.5510	−55.281	0.005	$7.48\times 10^{-3}$
